# Rapid Northward Spread of a Zooxanthellate Coral Enhanced by Artificial Structures and Sea Warming in the Western Mediterranean

**DOI:** 10.1371/journal.pone.0052739

**Published:** 2013-01-14

**Authors:** Eduard Serrano, Rafel Coma, Marta Ribes, Boris Weitzmann, María García, Enric Ballesteros

**Affiliations:** 1 Centre d'Estudis Avançats de Blanes-Consejo Superior de Investigaciones Científicas (CEAB-CSIC), Blanes, Girona, Spain; 2 Institut de Ciències del Mar-Consejo Superior de Investigaciones Científicas (ICM-CSIC), Barcelona, Spain; University of Vigo, Spain

## Abstract

The hermatypic coral *Oculina patagonica* can drive a compositional shift in shallow water benthic marine communities in the northwestern Mediterranean. Here, we analyze a long-term, large-scale observational dataset to characterize the dynamics of the species' recent northward range shift along the coast of Catalonia and examine the main factors that could have influenced this spread. The variation in the distributional range of *Oculina patagonica* was examined by monitoring 223 locations including natural and artificial habitats along >400 km of coastline over the last 19 years (1992–2010). Abundance of the species increased from being present in one location in 1992 to occur on 19% of the locations in 2010, and exhibited an acceleration of its spreading over time driven by the join action of neighborhood and long-distance dispersal. However, the pattern of spread diverged between artificial and natural habitats. A short lag phase and a high slope on the exponential phase characterized the temporal pattern of spread on artificial habitats in contrast to that observed on natural ones. Northward expansion has occurred at the fastest rate (22 km year^−1^) reported for a coral species thus far, which is sufficiently fast to cope with certain climate warming predictions. The pattern of spread suggests that this process is mediated by the interplay of (i) the availability of open space provided by artificial habitats, (ii) the seawater temperature increase with the subsequent extension of the growth period, and (iii) the particular biological features of *O. patagonica* (current high growth rates, early reproduction, and survival to low temperature and in polluted areas). These results are indicative of an ongoing fundamental modification of temperate shallow water assemblages, which is consistent with the predictions indicating that the Mediterranean Sea is one of the most sensitive regions to global change.

## Introduction

Human activities (e.g., overfishing, trawling, coastal development, deployment of man-made infrastructures, transportation, use of fossil fuels and pollution) are currently affecting marine ecosystems worldwide [1]–[3]. Two main phenomena – climate change and the introduction of alien species – appear to be increasing the rate of change in species distribution boundaries, making it possible to examine such changes on a decade time-scale [4]–[7]. The distributions of a wide range of taxa are expanding poleward [6], [8], and at the same time, coral reefs are undergoing rapid degradation due to increasing anthropogenic impacts, particularly climate change [9]–[12]. The poleward expansion of corals favored by increasing temperatures could compensate for the degradation of corals in their normal distribution range because of warming. However, it has been argued that coral species appear to be unable to disperse or adapt rapidly enough to cope with the current rate of change [12]–[15], but see [16]–[17].

The Mediterranean Sea is an optimal study site for early detection and characterization of the effects of global change on marine species ranges for several reasons. First, it is a semi-enclosed sea that is being affected by climate change at a faster rate than many other marine areas [18], and second, it is a hot spot for alien species, some of which are of tropical origin and have entered the Mediterranean via the Suez Canal [19]–[21]. An understanding of the spread dynamics of alien species and the main processes determining this spread are crucial for predicting future changes in their distributions in the context of global environmental change [4], [22]. Accurate predictions of the future distribution ranges of alien species that are able to change the structure and functioning of native ecosystems [23]–[25] are essential for determining their effects and for supporting management actions.

The most accurate records regarding the rate and the spatial pattern of the spread of alien species come from annual field surveys [26]. However, surveys repeated with high frequency in areas ranging from tens to hundreds of kilometers are rare, mainly due to the cost of monitoring large areas in detail. Thus, distributional data for most introduced species have low accuracy given that they comprise a relatively limited number of observations [27]–[28].

*Oculina patagonica* (De Angelis D'Ossat 1908) is an alien, non–lessepsian scleractinian zooxanthellate coral that presumably originated from the southwest Atlantic and was first recorded in the Mediterranean Sea in 1966 (when a single colony was found in the Gulf of Genoa, [29]). The unsolved problem is that the original description of *O. patagonica* is based on fossil material from Holocene deposits on the temperate coast of South America, and living specimens have not been found in this area. In the late 1970s, *O. patagonica* was abundant along ∼300 km of the southeast coastline of the Iberian Peninsula, indicating that this species had been present in this area for a long time when it was first recorded [30]–[32]. This species is also currently abundant on the coast of Israel where it was first recorded in 1993 [31]. During the last decade, *O. patagonica* has exhibited a 3-fold increase in certain littoral locations in southeastern Iberia [24], and isolated colonies have been found in several new areas throughout the Mediterranean (see [32] for a review), suggesting that *O. patagonica* is spreading geographically. The species presumably spread from the western basin to the levant basin via the intense intra-Mediterranean maritime traffic [31]. Both asexual dispersal in the form of polyp expulsion [33] and the release of gonads from colonies attached to ship hulls [29], which is favored because the species is capable of reproducing sexually when colonies are small [31], have been proposed as the primary vectors of invasive transport. Moreover, the species' ability to thrive and reproduce despite wide variations in temperature, salinity, UV radiation, turbidity and hydrodynamic conditions [31], [34] may have favored the species' spread throughout the Mediterranean.

In this study, the spread of *O. patagonica* was examined by monitoring >400 km of coastline over the last 19 years on the Catalan coast (northwestern Mediterranean). Whereas *O. patagonica* is abundant ∼30 km south of the Catalan coast ([35], authors' unpublished data), it was not recorded in the adjacent area to the north prior to 1992 [32]. Here, we analyze a long-term, large-scale observational dataset in order to characterize the dynamics of the species' recent northward range shift along the Catalonian coast and examine the main processes that could have influenced this spread.

## Materials and Methods

### Distribution surveys

**Figure 1:**
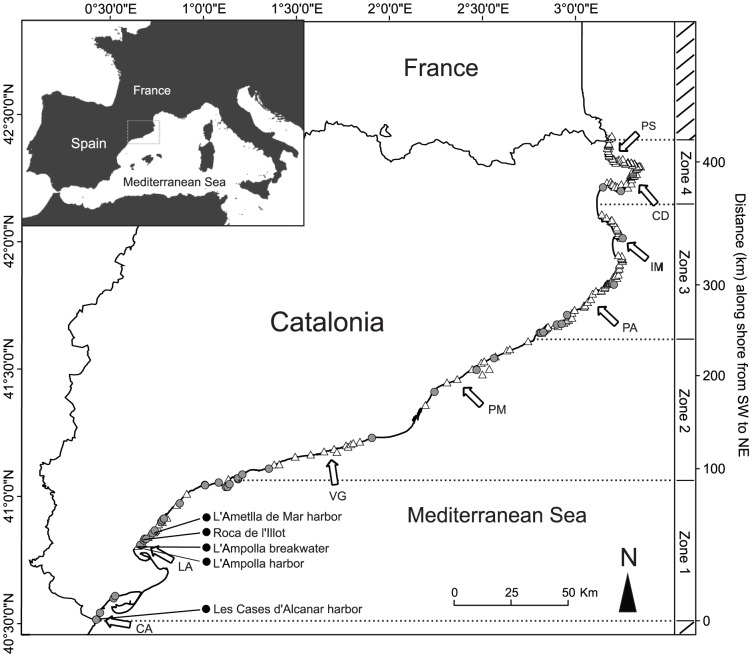
Map of the Catalan coast in the northeastern region of the Iberian Peninsula (northwest Mediterranean Sea). The positions of the 223 surveyed locations are indicated. Blank triangles (n = 180) represent surveyed locations in which *Oculina patagonica* was not found. Locations in which *O. patagonica* was found are represented by grey circles (n = 38; density <0,05 col. m^−2^) and black circles (n = 5; density >0,05 col. m^−2^). The linear coastal length from the southernmost location (in kilometers) is represented on the right-hand y-axis. Four zones of ∼100 km in length have been distinguished from the south (zone 1) to the north (zone 4) to examine the patterns in coral abundance. Sea surface temperature (SST) data have been obtained from two locations in each zone (indicated by white arrows): Les Cases d'Alcanar (CA), L'Ampolla (LA), Vilanova i la Geltrú (VG), Premià de Mar (PM), Port d'Aro (PA), Illes Medes (IM), Cadaqués (CD) and Port de la Selva (PS).

The study was performed along the Catalan coast in the northeastern region of the Iberian Peninsula (northwestern Mediterranean). The study area encompassed more than 400 km of coastline from Les Cases d'Alcanar in the south (40°31′N, 0°30′E) to Portbou in the north (42°26′N, 3°10′E, Fig. 1). A total of 223 locations were examined for the presence of the scleractinian coral *O. patagonica* over a period of 19 years (1992–2010) at intervals ranging between 1 and 2 years as part of a monitoring protocol designed to determine the arrival and occurrence of alien species in the shallow infralittoral zone. We surveyed both natural (n = 169) and artificial (n = 54) habitats. The natural habitats were rocky reefs, while the artificial habitats included man-made structures, primarily dikes, breakwaters and harbor walls. The mean linear distance between the examined locations was ∼2 km. At each location and sampling date, the presence or absence of the coral was determined through close examination of at least 1500 m^2^ of the sea floor using SCUBA.

The results of the systematic monitoring of *O. patagonica* were translated into absence/presence records over time at each location. The cumulative numbers of locations where the species was present were used to reconstruct the pattern of its spread 36–37. The initial detection of the coral was unequivocal for colonies larger than 5–10 cm in diameter; however, smaller colonies could be overlooked. It was noteworthy that over the study period, the species did not disappear from any location after its initial detection there. Thus, although the initial detection underestimates spread rate of *O. patagonica*, this species persistence in all locations where it was found over the study period indicates that the detection of small colonies provides a fair estimation of when this species became established. This pattern is most likely related to the size-dependent nature of coral mortality [38]–[39]. To reduce the bias introduced by the fact that not all locations were examined every year, gaps in the data obtained at a particular location after the coral was first detected were marked as the species being present based on species persistence. Then, to examine the pattern of *O. patagonica* spread over the study period, we calculated the cumulative number of locations where the species was present and expressed this as a percentage of the total number of locations.

Regression models were fitted to the standardized cumulative number of locations where the species was present over time (termed standardized spread curves [37], [40]), and the slope of the linear regression of the log-transformed cumulative number of locations was used as a measure of spreading success [37], [41]. A standardized spread curve was calculated separately for each of the two habitat types (i.e., natural and artificial).

The different phases of the spreading process (lag, exponential and saturation; [42]) were examined in the standardized spread curves. The beginning of the exponential phase was defined as the year in which the parameters of the standardized spread curve changed. This phase was detected using a maximum likelihood estimation of classic regression parameters for partitioned models [43]–[44]. The shift was set at the F-value of maximum significance (p<0.0001). The coefficient of the linear regression of the cumulative number of locations after the lag phase over time was used as a measure of the rate of the exponential phase. Differences in the slopes of the overall standardized spread curves and in the slopes of the exponential phase were tested using the homogeneity of slopes test [45].

We examined whether the northern limit of the distribution of *O. patagonica* varied over time. The linear correlation coefficient of the northern distribution limit of the species over time was used to estimate the expansion rate (km year^−1^).

### Current distribution and abundance (2008–2010)

Between 2008 and 2010, field surveys also included measurements of all coral colonies and an annotation of the depth of each colony. Within the study area, the *O. patagonica* colonies displayed a predominantly encrusting growth form with a circular-ellipsoidal shape. A colony was defined as any distinct, single coral skeleton with living tissue. A specimen divided by partial mortality into separate patches of living tissue that was morphologically still one entity was considered as a single colony. Neighboring colonies in close proximity, which were found on several occasions, were measured as distinct. The surface area of the colonies was estimated by means of *in situ* measurements of the longest dimension of the colony (length, L) and its perpendicular axis (width, W) to the nearest millimeter using a ruler. The surface area (S, cm^2^) was calculated using the formula S = π[(L + W)/4]^2^ according to [31].

Two methods were used to estimate coral abundance on hard substrata between 0 and 10 m in depth (or until the maximum depth when the depth was <10 m), which is the depth range within which *O. patagonica* has been reported to be the most abundant [31]–[32, authors' observations]. When the species was scarce (hereafter, present), we assessed its abundance by counting and measuring all colonies present on 1500 m^2^ of the seabed using SCUBA. When the species was abundant (hereafter, populations), we conducted three replicates of randomly localized 40 m^2^ transects (40×1 m, Fig. S1). Only colonies with at least 50% of their surface area lying within the belt transect were counted and measured to avoid boundary effect biases in the spatial sampling method [46]. Coral abundance data were standardized based on the area surveyed, and the species density and percent cover values were calculated for each location.

We utilized the standard error (SE) sample size function to determine the minimal sampling area needed to assess the density and percent cover of the coral populations [47]–[48]. Preliminary sampling to obtain coral abundance estimates was conducted at one of the study locations (Les Cases d'Alcanar) by examining an area of 200 m^2^ using 5 m^2^ belt transects (5×1 m) set randomly on the substrate between depths of 2 and 4 m.

We partitioned the entire coast into four similar zones (each ∼100 km of coastal length, Fig. 1) to identify the mechanisms that contribute to the variation in the abundance of *O. patagonica* colonies across the study area. The number of locations where the species occurred was calculated separately for each zone to examine whether the coral exhibited an identifiable pattern along the coast. The proportion of locations where the coral occurred in each of the four zones was calculated separately for the two habitat types (i.e., natural and artificial).

### Temperature

Temperature has long been considered to be one of the main environmental factors controlling coral species distributions (e.g., [49]–[50]). We obtained a sea surface temperature (SST) time series from 8 locations distributed along the ∼400 km Catalan coast (Fig. 1). The cumulative distances between the locations at which temperature was examined, ordered from south to north, are indicated in Table S1.

Daily mean SSTs were obtained from January 2003 to December 2010 from satellite measurements performed by the MODIS (aqua) sensor system (http://oceancolor.gsfc.nasa.gov/), which were made available as “Ocean Level-2” HDF data by NASA's Goddard Space Flight Center. HDF files were read and processed using Matlab R2009a (MathWorks Inc., Natick, MA, USA). In the analysis, we considered only high-quality temperature readings (flag values of 0 or 1), and we discarded less reliable readings (flag values of 2 or 3). The suitable SST readings used in our analyses corresponded to the daily means in a 9-km^2^ area centered on the geographic coordinates indicated in Table S1.

Over the SST study period, the mean annual temperature and mean annual 95^th^ and 5^th^ percentiles were determined for each location along the Catalan coastline. On the basis of the SST data recorded daily, we also calculated the number of days that the temperature was above or below different thresholds specifically chosen because of their biological consequences (i.e., relying on previous field observations and/or thermo-tolerance experiments performed on *O. patagonica* [51]–[52]). To examine whether exposure to summer conditions (>18°C, [53]) and to upper sub-lethal temperatures (24 and 26°C) could affect the target species, we determined the day in spring/summer on which the SST reached ≥18°C, ≥24°C, and ≥26°C and the day in late summer/fall on which it dropped below each temperature. To avoid any bias introduced by short-term temperature oscillations, we determined that after the date on which the SST reached ≥X°C, the daily mean SST values had to remain ≥X°C on at least 80% of the days throughout the following two weeks (i.e., 11 of 14 days). These temperature thresholds were used to assess the temperature regimes among 8 locations distributed along the 400 km of the Catalan coastline.

Due to the assumptions made by the Pearson's correlation analyses, we estimated the confidence intervals when the n value was <12. The 95% confidence intervals were calculated using bias-corrected bootstrapping with 1,000 resamples, which were considered significant when the confidence limits did not include zero [54]. All analyses were computed using STATISTICA 7.0, except for the bootstrapping analyses, which were implemented with R [55].

## Results

### Current distribution along the Catalan coast

We found that *O. patagonica* was present in wide areas along the study coast at a depth range of 0–10 m. The species was rarely observed below this depth, although a few colonies were found down to 28 m depth.

**Figure 2:**
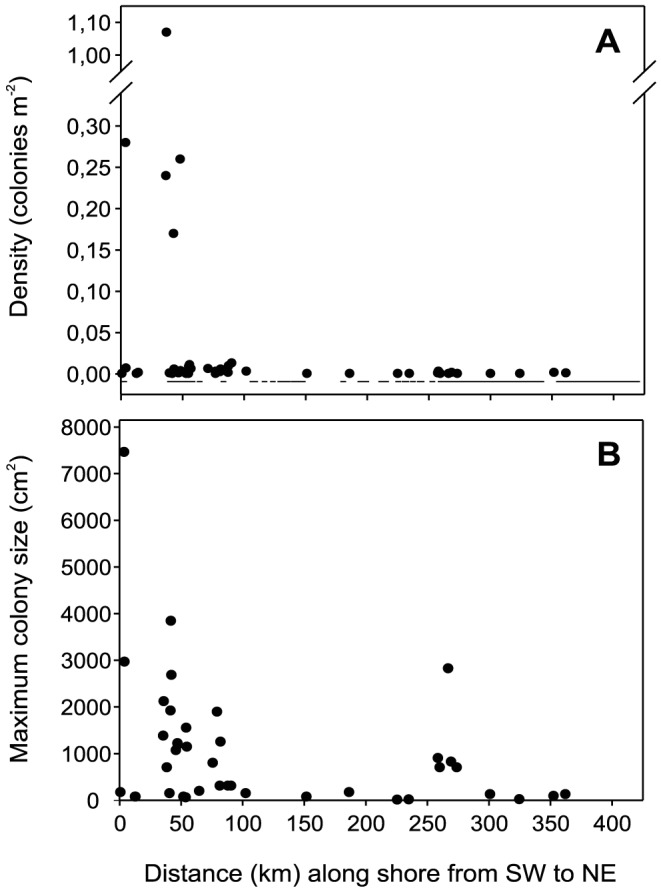
Density and maximum colony size of *Oculina patagonica* along the Catalan coast. A) Current coral density (colonies m^−2^) in the locations at which the species was observed along the linear length of the Catalan coast (km). Horizontal lines below the zero line indicate locations at which *O. patagonica* was not encountered. B) Maximum colony size (cm^2^) in the locations at which the species was observed in 2010.

*O. patagonica* abundance decreased from south to north, and significant populations (defined as those with a density of >0.05 colonies m^−2^) were found in 5 locations, all of which were situated in the southernmost zone of the studied region (Fig. 2a). At all other locations where this species was observed, it occurred at a density of <0.05 colonies m^−2^. Occurrence of the species at densities of <0.05 colonies m^−2^ was defined as presence. Four of these populations were located in artificial habitats, and one was located in a natural habitat. The density of *O. patagonica* populations in the artificial habitats ranged from 0.24 to 1.07 colonies m^−2^ (0.46±0.20 colonies m^−2^, mean ± SE), which was approximately three-fold higher than the density observed in the single natural habitat (0.17±0.02 colonies m^−2^, mean ± SE). The percentage of the sea floor occupied by the species (percent cover) in artificial habitats ranged from 0.51 to 5.51% (2.48±1.13%, mean ± SE), which was approximately two-fold higher than that of the population in the single natural habitat (1.35±0.48%, mean ± SE). The species density at the 38 locations where it was present was also two-fold higher in artificial habitats than in natural habitats (0.0042±0.0009 vs. 0.0020±0.0004 colonies m^−2^, respectively; mean ± SE; One-way ANOVA, F_1,36_ = 4.6563, p = 0.0377). Of the total number of *O. patagonica* colonies measured (n = 670), 80% were found in artificial habitats, and 20% were located in natural habitats.

In accordance with the species abundance, the maximum colony size decreased from south to north. The locations with the largest colony sizes (>1000 cm^2^) were mainly found in the southernmost zone of the region (Fig. 2b). The maximum colony size was less than 1000 cm^2^ at all other locations, with the exception of the location where a discrete long-distance dispersal event occurred in the year 2000 (see below). The maximum colony size did not vary between the artificial and natural habitats (1156±363 vs. 925±255 cm^2^, mean ± SE, respectively; One-way ANOVA, F_1, 38_ = 0.2623, p = 0.6115).

**Figure 3:**
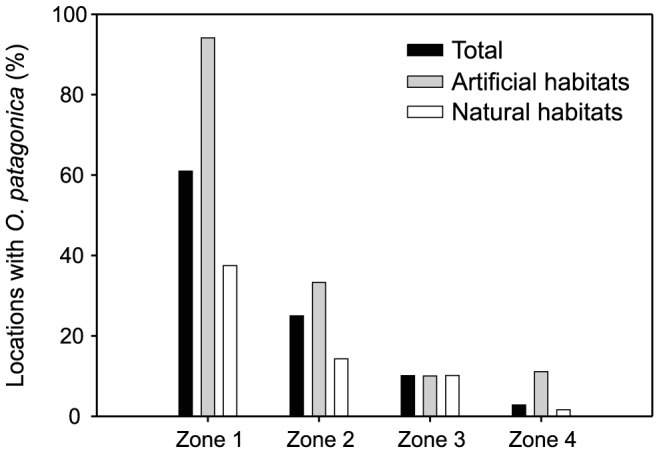
Proportion of locations where *Oculina patagonica* was observed in four zones along the Catalan coast. See Fig. 1 for a description of the four zones. The proportion of locations at which the species was encountered in 2010 is given based on the total number of locations and the habitat type (i.e., natural and artificial).

The proportion of locations where the species occurred decreased steadily from 61% in the southernmost zone to 3% in the northernmost zone (Fig. 3), although this pattern differed depending on habitat type (i.e., natural and artificial). In artificial habitats, the proportion of locations where the species occurred decreased steadily from 94% in the southernmost zone to 11% in the northernmost zone (Fig. 3), while in natural habitats, the proportion of locations where the species occurred decreased steadily from 38% in the southernmost zone to 2% in the northernmost zone (Fig. 3).

### Temporal variation of species distribution

**Figure 4:**
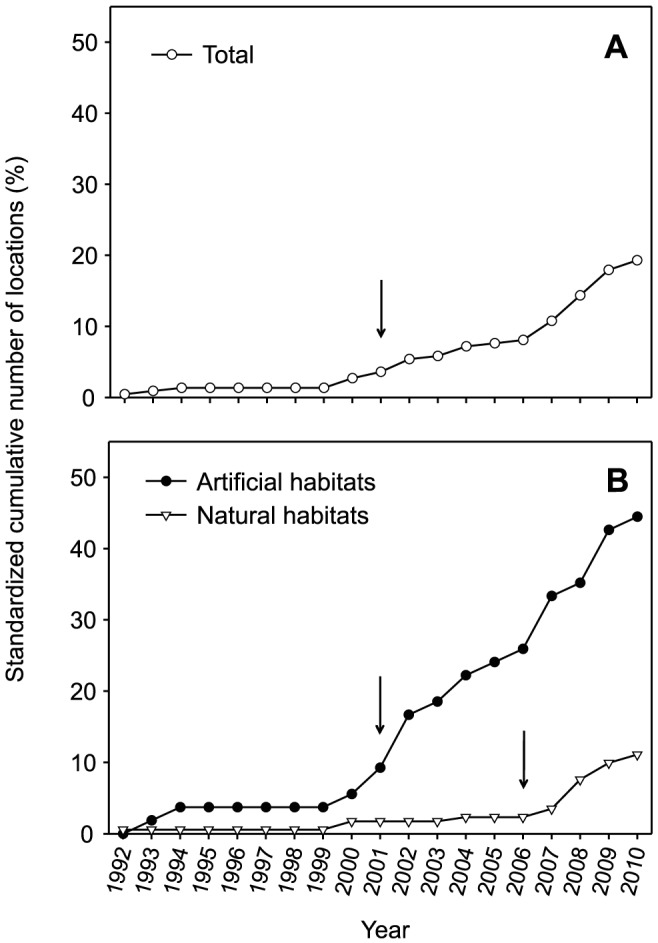
Standardized spread curves of *Oculina patagonica* from 1992 to 2010 along the Catalan coast. The standardized cumulative number of locations where the species occurred is given for (A) the total data and (B) the habitat type (i.e., natural and artificial). The beginning of the exponential phase is indicated by an arrow (see Methods).

A solitary colony of *O. patagonica* was discovered for the first time in a natural habitat located in the southernmost area of the Catalan coast (Les Cases d'Alcanar) in 1992. A year later, an *O. patagonica* colony was recorded for the first time in an artificial habitat. From 1993 until 2010, the number of locations at which the species was present along the Catalan coast increased exponentially (Fig. 4a). In 2010, the species was present in 19% of all explored locations (43 of the 223 explored locations, Fig. 4a). However, this pattern diverged markedly between the two distinct habitat types; the species was observed in 44% of the artificial habitats and only in 11% of the natural habitats (Fig. 4b).

**Figure 5:**
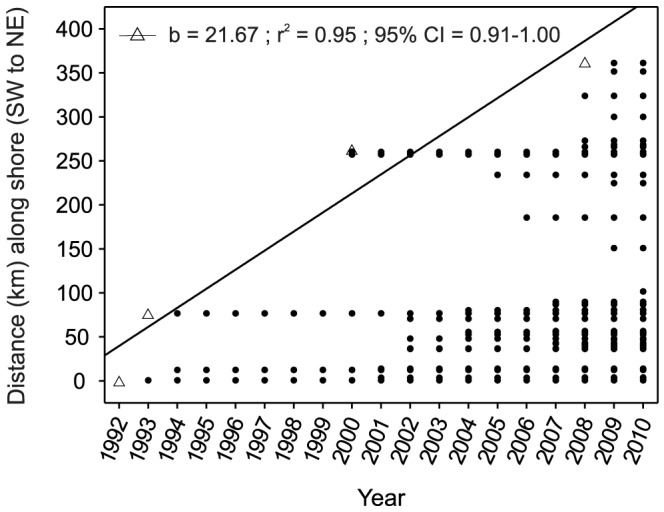
*Oculina patagonica* records obtained on the Catalan coast throughout the study period (1992–2010). Records of the coral occurrence (full circles) throughout the Catalan coastal length (km) from 1992–2010 illustrate the spreading of the species over time. Blank triangles indicate the change in the northern latitudinal limit over time. The expansion rate (km year^−1^) was calculated as the slope of the Pearson product-moment correlation between the change in the northern latitudinal limit of the coral species and time (n = 4). 95% CI: 95% confidence interval calculated with bootstrapping.

Exponential regression models provided the best fit to the increase in the cumulative number of locations over time. The cumulative number of locations where *O. patagonica* has been found over the 19-year study period increased ∼1% every year (Fig. 4a) and showed a pattern of geographic spreading (Fig. 5). The overall pattern of geographic spreading was characterized by a lag phase of 9 years and a steady increase since 2001 (Fig. 4a, Table 1). However, this pattern diverged between natural and artificial habitats (Fig. 4b); the duration of the lag phase for the natural habitats was approximately twice that of the lag phase associated with artificial habitats (Table 1). The number of locations at which the species was present at the end of the lag phase was low for both habitat types (2–9% of the examined locations), indicating that the exponential phase began immediately after *O. patagonica* established foci in a particular region (Table 1). Throughout the entire study period, the slope associated with the artificial substrata was slightly higher than that associated with the natural substrata. This effect was accentuated in the exponential phase, during which the slope for the artificial substrata was significantly higher than that for the natural substrata (F = 29.1849, p<0.05, Table 2). Between 2001 and 2006, the overall slope of the exponential phase was reduced because the natural habitats were still in the lag phase. After 2006, the slope exhibited an increasing trend because both habitats were in the exponential phase (Fig. 4, Table 2). Therefore, the species is spreading faster among artificial habitats than natural habitats.

**Table 1:** Characteristics of the standardized spread curves of *Oculina patagonica* along the Catalan coast.

		First detection	Duration of L	Beginning of E		
	N	in Catalan waters	(years)	Year	Loc. no.	% N
Total	223	1992	9	2001	8	3.59
Artificial	54	1993	8	2001	5	9.26
Natural	169	1992	14	2006	4	2.34

A standardized spread curve is given for the total data and the distinguishing habitat type (i.e., artificial and natural). N: number of studied locations; L: lag phase; E: exponential phase; Loc. no.: number of locations; %N: percentage of the cumulative number of locations at which the species was observed at the beginning of the exponential phase.

**Table 2:** Spread rate of *Oculina patagonica* along the Catalan coast.

	_______Entire study period _________					______Exponential phase______				
	Slope (b)	SE of b	r^2^	p-value	N	Slope (b)	SE of b	r^2^	95% CI	N
**Total**	0.08	0.00	0.96	<0.0001	19	1.71	0.19	0.91	(0.83–0.98)	10
**Artificial**	0.08	0.01	0.94	<0.0001	18	3.74	0.20	0.98	(0.94–1.00)	10
**Natural**	0.07	0.01	0.88	<0.0001	19	2.40	0.28	0.96	(0.90–1.00)	5

Coral spread rate for the 19-year study period (left side of the Table), is expressed as the slope of the linear regression of the log-transformed standardized cumulative number of locations where the species was present versus time. For the exponential phase (right side of the Table), the spread rate is expressed as the slope (b) of the linear regression of the standardized cumulative number of locations where the species was present versus time. In each case, estimates were calculated separately for the total data and the distinguishing habitat type (i.e., natural and artificial). SE: Standard error. N: number of years. 95% CI: 95% confidence interval calculated with bootstrapping.

### Latitudinal distribution limit and spreading patterns

**Figure 6:**
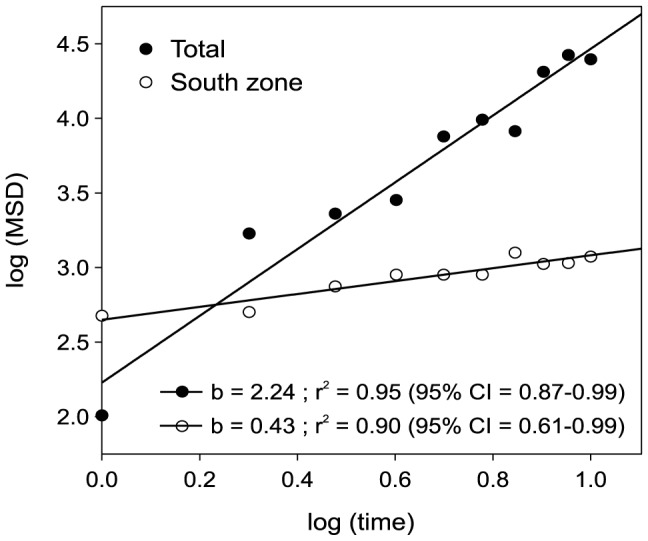
Patterns of *Oculina patagonica* dispersal. Mean square displacement (MSD) between the locations at which the coral species was found after the lag phase. The dispersal of the species during the exponential phase of spreading is provided separately for the overall distribution (Total) and for the southern zone (0 to 100 km coastal length). MSD is proportional to t*^α^* where t is time and *α* is the scaling exponent. 95% CI: 95% confidence interval calculated with bootstrapping.

The northern limit of the distribution of *O. patagonica* varied over time, exhibiting a northward geographic spread (Fig. 5). Over the 19-year study period, this limit spread 361 km northward at a mean rate of 21.67±3.70 km year^−1^ (mean ± SE). The presence of *O. patagonica* in the littoral zone underwent lag phases over time (see above), and some long-distance dispersal events ranging between 76 and 182 km (120±32 km; mean ± SE, Fig. 5) created new invasion foci. After the lag phase, the mean square displacement (MSD) between the locations at which the species was present over time showed a scaling exponent of *α*∼2 (MSD is proportional to *t^α^*, where *t* is time and *α* is the scaling exponent, Fig. 6), which indicates an acceleration of spreading over time [56]. This accelerating pattern is strongly driven by a few intermittent long-distance dispersal events. To distinguish local dispersal from long-range dispersal, we estimated the MSD in the southern region of the coral's distribution (first ∼100 km of coastline, Fig. 5), which is separated from the northern region by ∼50 km of coastline where the species is not present (Fig. 2a). After the lag phase (in 2001), the MSD of the southern region (0–150 km) showed a scaling exponent of *α* = 0.43, indicating a decline of spreading over time (Fig. 6).

### Latitudinal temperature analysis

**Figure 7:**
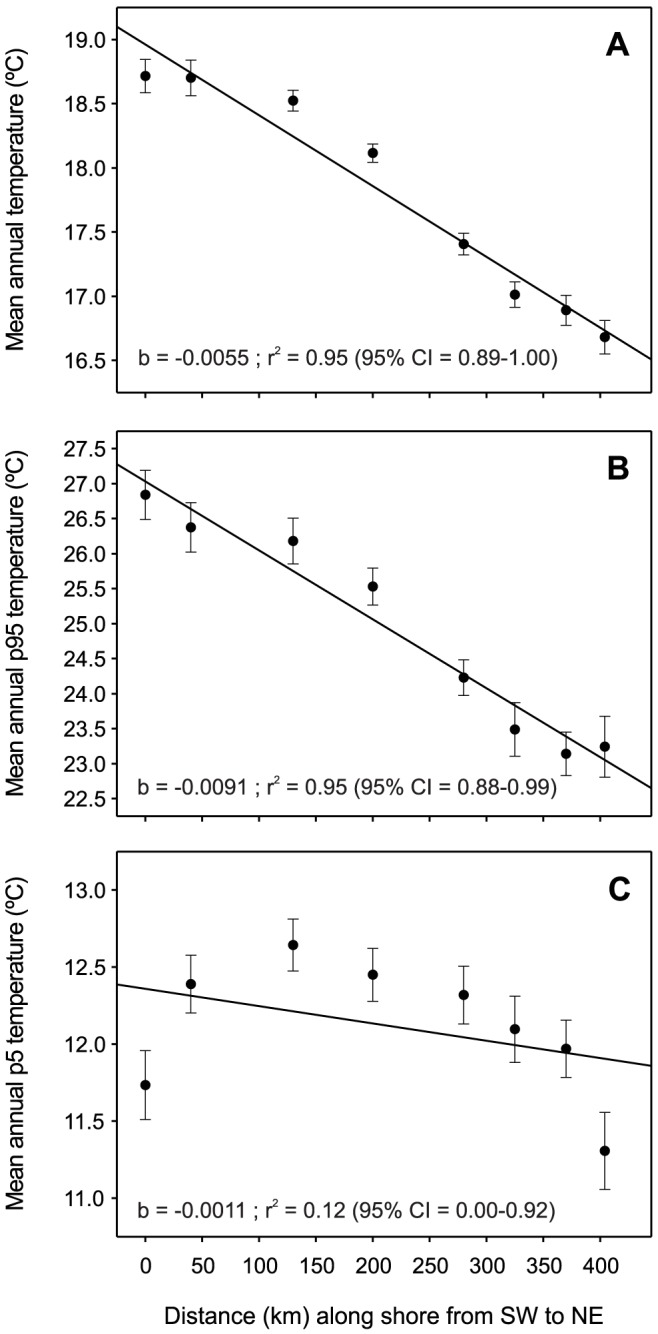
Sea surface temperature (SST) obtained from NASA satellite measurements over the last 8 years (2003–2010). See Fig. 1 and Table S1 for a description of the studied locations (n = 8). A) Mean annual temperature; B) mean annual temperature 95^th^ percentile (p95); C) mean annual temperature 5^th^ percentile (p5). Pearson product-moment correlation between the distance (km) along the shore from SW to NE (see Fig. 1) and the temperature along the Catalan coast. 95% CI: 95% confidence interval calculated with bootstrapping.

The SST along the studied coastline displayed a northward latitudinal decrease in the mean annual temperature of 0.55±0.05°C/100 km, ranging from 18.72±0.13°C (mean ± SE) in the south (CA) to 16.68±0.13°C in the north (PS) (Fig. 7a). The mean annual temperature did not vary over 8 years according to the available satellite data (2003–2010) at any of the 8 examined locations (data not shown). This is most likely related to the short-term oscillatory pattern in northwest Mediterranean temperatures [57]. The mean annual 95^th^ percentile (p95) decreased by 0.91±0.09°C/100 km with increasing latitude, ranging from 26.84±0.35°C (mean ± SE) in the south (CA) to 23.24±0.44°C in the north (PS) (Fig. 7b). The mean annual 5^th^ percentile (p5) did not vary significantly due to the local temperature conditions of the southernmost location (CA), which is subjected to discharge from the Ebro River (Fig. 7c). However, when this local effect was not considered, p5 exhibited a similar significant pattern of a northward decrease [0.26±0.09°C/100 km, r^2^ = 0.62, 95% confidence interval = 0.41–1.00 (bootstrap analysis), n = 7]. We are aware that the n value was inappropriate for the Pearson's correlation method, but bootstrapped confidence intervals provided an estimate of the probability that the true correlation coefficient do not include zero.

We calculated the number of days on which the SST was ≥18°C as a temperature indicator of warm conditions that favor *O. patagonica* growth [52] as long as the upper sublethal threshold is not reached [51]. The mean annual number of days on which the SST was ≥18°C exhibited a decreasing latitudinal pattern from south to north, ranging from 188±4 days year^−1^ in the south (CA; mean ± SE) to 143±5 days year^−1^ in the north (PS) (Table 3). Additionally, the mean annual number of days on which the SST was ≥18°C was 24% lower in the north (PS-CD) than in the south (CA) and exhibited an average decrease of 12.35±1.13 days year^−1^ (slope ± SE) for each 100 km northward (Table 3). We calculated two indicators of elevated temperature conditions that have been documented to cause colony damage and decrease the growth of *O. patagonica* after a certain period of exposure: (1) the annual number of days on which the SST was ≥24°C [52] and (2) the annual number of days on which the SST was ≥26°C [51]. The mean annual number of days on which the SST was ≥24°C exhibited a northward decrease, ranging from 90±4 (mean ± SE) days in the south (CA) to 8±5 days in the north (PS). The mean annual number of days on which the SST was ≥24°C was 91% lower in the north (PS) than in the south (CA) and exhibited an average decrease of 22.41±2.05 days year^−1^ (slope ± SE) for each 100 km northward (Table 3). The mean annual number of days on which the SST was ≥26°C exhibited a northward decrease of 7.21±1.15 days/100 km (Table 3), ranging between 31±7 days in the south (CA) and 0 days in the north (PA and upper northern locations). Colony damage related to high temperature was not observed during exposure to summer temperature conditions from 2008–2010 at the southern location (LA), where the SST was ≥24°C for 61–82 days year^−1^ and was ≥26°C for 0–28 days year^−1^ (colonies monitored seasonally over the period 2008 and 2010, authors' unpublished data). In contrast, colony damage that was apparently related to exposure to low temperature during the winter was observed every year (authors' unpublished data).

**Table 3:** Sea surface temperature thresholds along the Catalan coast.

SST indicator	slope (b)	SE of b	r^2^	95% CI	N
no. of days ≥18°C	−0.12	0.01	0.95	(0.86−1.00)	8
no. of days ≥24°C	−0.22	0.02	0.95	(0.89−0.99)	8
no. of days ≥26°C	−0.07	0.01	0.87	(0.71−0.98)	8

Pearson product-moment correlation between coastal length (km from south to north, see Fig. 1) and sea surface temperature (SST) indicators (mean annual number of days on which the temperature was above 18°C, 24°C and 26°C per year from 2003 to 2010) along the Catalan coast. SE: standard error of the slope (b). N: number of locations where SST was measured along the Catalan coast. Temperature values were obtained from satellite data, see Methods. 95% CI: 95% confidence interval calculated with bootstrapping.

## Discussion

Small cryptic alien species may be present at low numbers for years before they are detected [58]. However, this is most probably not the case for the alien coral *O. patagonica* in Catalonia because (1) it occurs mainly in the shallow infralittoral zone, which has been well studied in previous taxonomic surveys during the 1970s and early 1980s [59]–[63]; (2) it achieves a large colony size (>50 cm in diameter); (3) it is easy to identify and has previously been detected in other Mediterranean areas [29]–[30]; and (4) the Catalan coast was regularly explored (every 1–2 years) at a high resolution (mean distance between locations of 2 km) throughout the entire study period (1992–2010) with the specific objective of detecting alien species in this habitat. We therefore conclude that *O. patagonica* should be a recent addition to the studied coast.

Although the regular monitoring of *O. patagonica* has allowed us to characterize the dynamics of its spreading, the initial detection of the species at a sampling station did not necessarily correspond to the date of its arrival because we were not able to detect very small colonies with one polyp or a very low number of polyps. In fact, according to Lodge [64], the proportion of species introduced outside of their original range that become established is generally low for a wide variety of reasons, and the time of establishment is ecologically more relevant than the time of first arrival. The fact that *O. patagonica* did not disappear from any of the locations where it had been detected over the 19-year monitoring period indicates that our time of initial detection is a conservative estimate of the time of establishment.

### Spreading dynamics

The use of a standardized spread curve has allowed us to characterize a 9-year lag period, during which the spreading rate was much lower than that in the exponential phase. After the establishment of the species at a particular location, its colonies begin to grow and reproduce. Thus, the observed lag phase could be related to the fact that broadcast spawning corals, such as *O. patagonica* [31], rely on an external fertilization process (in which gametes are released into the water column for dispersal) that requires a critical density of adults to ensure successful fertilization [65]–[68]. Additionally, the species is a gonochoric coral, and a skewed sex ratio has been documented at certain locations with low coral abundance [31]. Therefore, Allee effects could be contributing to the observed lag phase in this species' spreading dynamics. Surprisingly, the lag phase of this coral species is within the range documented for most invasive benthic macroalgae (5–10 years, [28]), which is likely attributable to the particular biological characteristics of *O. patagonica* (see below). The beginning of the exponential phase occurred when *O. patagonica* was present at 3.6% of the total number of investigated locations, indicating that the exponential phase began shortly after the species established foci in the region.

The observed northward spread of *O. patagonica* across the study area coupled with the northward decrease in coral abundance and maximum colony size provides strong evidence of a recent and ongoing poleward expansion. The MSD estimate for the southern region of *O. patagonica* distribution, which is indicative of the pattern of dispersal to nearby locations, showed a decrease in spreading over time [56]. This finding is consistent with the fact that the species faces less favorable conditions while spreading northward [69]–[70]. However, the MSD estimate for the overall distribution indicated a pattern of spreading acceleration over time. The differences in the patterns emerging from the two estimates are based on the occurrence of a few intermittent long-distance dispersal events (i.e., punctuated dispersal, [71]). The occurrence of intermittent long-distance dispersal events exerted a large effect on the overall pattern of spreading of the species by promoting a regime shift from a decreased to an accelerated pattern of spreading over time. Thus, the rapid spreading of *O. patagonica* appears to have been accomplished through the interplay of two spatial scales of dispersal: neighborhood dispersal and long-distance dispersal interspersed with lag periods. This rapid spreading is consistent with a large increase in the spreading rate that may be caused by a small number of long-distance dispersal events, as documented in other observed and modeled populations [72]–[74].

Various tropical corals appear to be shifting their distributions to higher latitudes in the western Atlantic (6–10 km year^−1^; [75], *sensu* [76]), the Indo-Pacific [77] and the western Pacific (2–14 km year^−1^; [17]). The northward spreading rate of *O. patagonica* observed in this study (22 km year^−1^) is the highest reported for a native or alien scleractinian coral to date and is consistent with the recently documented presence of isolated colonies of this species in distant zones throughout the Mediterranean [32].

Gametogenesis has been observed in colonies from the study area (authors' unpublished data). Dispersal to nearby areas can be achieved through the dispersion of planula larvae and via the polyp expulsion mechanism [33]. Long-distance dispersal can also be achieved through planula dispersion mediated either by currents or ballast waters as previously suggested [29], [32]. However, the capacity of this species to settle on floating objects ([31], authors' observations) and to thrive in harbors and polluted areas [78] suggests that drifting on artificial substrata may be another vector favoring dispersal. Long-distance dispersal mediated by floating objects has been shown to contribute to the spreading of rapid colonizer species of coral [79]–[82]. Although sea surface circulation along Catalonia typically occurs in a southwestward direction [83], strong southern winds also occur regularly [84], which may help to explain the observed northward spreading of *O. patagonica* (Text S1). This spreading, via the above-mentioned dispersal mechanisms, indicates the high capacity of this species for dispersal, recruitment, growth and survival, which is consistent with its biological characteristics (see below).

### Causes of northward expansion

Successful establishment in a new area is dependent on the physical environment and the life-history traits of the species together with biological interactions [85]–[86]. However, the response of a species to environmental change could affect the outcome of biotic interactions such as competition and predation [87]–[90]. For example, when *O. patagonica* comes into contact with a bryozoan species, the competitive outcomes vary depending on whether the coral is affected by bleaching [91]. Competition between macroalgae and corals plays a significant role in determining the composition of benthic communities [10], [92]–[93]. Macroalgae are dominant in shallow habitats in temperate ecosystems [69], [94]–[95], and this dominance is especially evident in the Mediterranean [96]–[98]. Although macroalgae have been strongly affected during the last century due to habitat alteration along the Catalan coast, the shallow infralittoral zone in which the species dwells is still spatially limited [99]–[100]. In the northwest Mediterranean Sea, extreme physical disturbances and/or the high sea urchin abundance are the main mechanisms that create significant open spaces in an otherwise spatially limited macroalgal community [101]–[102]. Although the Catalan coast was affected by several large storms (defined as storms with waves ≥3 m) during the study period, only the storm of December 2008 was sufficiently large to cause relevant changes in the availability of open spaces [103]. Thus, the observed pattern of northward spreading cannot be attributed to a variation in the occurrence of physical disturbances. With regard to herbivory pressure, an increase in sea urchin abundance has been shown to increase the availability of open spaces and facilitate an increase in the abundance of coral species by reducing the competitive dominance of algae [24], [104]–[105]. However, sea urchin abundance did not increase over the course of the study, and in some areas, it decreased due to legal and illegal fishing [106]–[107]. Hence, the northward spread also cannot be attributed to a change in sea urchin abundance. Therefore, we conclude that physical and biotic disturbances have not varied much over the last twenty years, and although some changes in the composition of macroalgal communities have been detected, no major structural changes have occurred.

Although the main reasons for the northward expansion of *O. patagonica* are difficult to identify, the observed pattern of spread suggests some factors that must have played a relevant role in promoting the spread of this species. The high proportion of *O. patagonica* in artificial habitats in contrast to natural habitats (see Results) indicates that artificial habitats represent important places that foster the abundance and dispersal of this species. Space availability is the main initial difference between natural and artificial habitats. Assemblages flourishing in artificial habitats become similar to those of adjacent rocky bottoms over time; however, even after many decades, the assemblages do not usually resemble each other because artificial habitats are characterized by a higher space availability and a lower species richness, both of which are factors that increase habitat invasibility by primary space occupiers such as *O. patagonica* [22], [71], [108]–[110]. Thus, space availability plays a crucial role in explaining the differential spreading observed between the two types of habitats, which is consistent with previous results showing that open spaces created by the grazing activity of sea urchins enhances the settlement and survival of *O. patagonica* [24].

The deployment of coastal infrastructure causes ecological impacts by altering water flow, light penetration and sedimentation rates in shallow coastal waters [111], which negatively affects the growth of scleractinian corals [112]. However, field studies and laboratory experiments have revealed that *O. patagonica* has an exceptional capacity to grow under a wide range of environmental conditions [31]; as such, it is less affected than many native species by the above-mentioned side effects of artificial habitats. Therefore, the increased space availability on artificial substrata and the increasing deployment of artificial marine habitats in Catalonia (Fig. S2) appear to be the main causes of the observed differences in the spreading dynamics of the species between the two habitat types. Artificial habitats may be acting as corridors that facilitate the expansion of this species. Thus, although *O. patagonica* has been known to occur south of Catalonia since the 1970s [30]–[31], the fact that the species did not reach the study area until the early 1990s, despite the presence of significant amounts of artificial substrata prior to that time and several large populations within <30 km of the first discovery site on the Catalan coast (some with >20% cover [35], authors' unpublished data), is indicative of a change in the spreading dynamics at the end of the 1990s and throughout the 2000s, which may be related to temperature.

Water temperature is among the main factors affecting the distribution of corals [73], [113]–[114] and has been increasing over the last decades in the northwest Mediterranean [18]. Temperature has been hypothesized to limit coral reef growth directly by reducing the capacity of corals to achieve successful reproduction and recruitment [115]–[117], reducing coral growth [118] and causing coral mortality as a result of cold temperature episodes [119]. Damage to *O. patagonica* colonies in winter (partial mortality) occurs in the southern locations of the study area (authors' unpublished data) and is likely to be related to cold temperatures as previously documented [52]. Despite this damaging effect, *O. patagonica* is especially abundant in these southern areas; therefore, current winter temperature (11.7°C, the lowest 5^th^ percentile except for the km 400) is not preventing the northward expansion of the species. The results raise the questions of: a) whether extreme cold temperature events may have prevented the northward establishment of this coral in the past, and b) whether the current 5^th^ temperature percentile at km 400 (11.3°C) may prevent further northward spread of the species.

A long-term data series (1974–2010) obtained *in situ* at 0.5 m depth was used to estimate the rate of the mean annual temperature increase on the Catalan coast (0.032°C year^−1^) [18]. Based on this estimation, the mean annual temperature has increased ∼0.6°C over the 19-year study period. Additionally, the growing season (number of days with SST ≥18°C) has been lengthened by 0.76 days year^−1^ due to the warming pattern; therefore, it has increased by ∼14 days over the study period (1992–2010; authors' unpublished data). This corresponds to a 10% increase of the period in which the species growth is 2 folds higher than that at lower temperature [52]. However, the warming pattern also increases the occurrence of extreme high temperature episodes [53], [120], and previous experiments have shown that high temperatures affect *O. patagonica* [51]–[52], [121]. Interestingly, we have not observed coral bleaching and/or partial mortality in relation to high temperatures in the region; thus, the current high summer temperatures have not caused conspicuous harm to the species in the study area. This finding is consistent with the fact that the upper sea temperature thresholds that were documented to cause damage to this coral in Israel (i.e., >36–44 days above 26°C, [51]) have not been reached in the study area. Thus, the current pattern of seawater temperature increase favors coral growth by extending the growing period of the species, as has been documented in other species from terrestrial ecosystems [122]. The lack of apparent damage to the colonies submitted to the highest 95^th^ temperature percentile (26.8°C) indicates that, if the current pattern of sea warming is maintained, lengthening of the species' fast growing period will continue to favor the species growth during the following decades because most of the study area is far below the value of this percentil. *Oculina patagonica* exhibited a rapid increase after 1999. The cause of this timing can not be addressed, however, it coincided with the time period in which warming accelerated in the Eastern Mediterranean Sea causing an amplification of the entry of alien species [7].

Although successful establishment is affected by environmental conditions and interspecific interactions, the expansion of a population mainly depends on growth and dispersal and, consequently, on the life-history characteristics of each species [58], [123]. Therefore, biological traits that are characteristic of an opportunistic colonizer may have contributed to the observed pattern of *O. patagonica* expansion; these traits include (a) the ability to function as a facultative zooxanthellate species; (b) the capacity to reproduce both sexually and asexually and to be fertile at an early age (1–2 years, [31], [33]); (c) the ability to survive extreme environmental conditions (i.e., in tide pools, at temperatures of 10–40°C and at salinities of 28–50‰, [31]); and (d) the capacity to survive and grow in harbors, polluted areas, and areas affected by severe sand scouring as well as on undisturbed natural rocky bottoms [78].

Although environmental conditions are among the major determinants that shape species distribution ranges [113]–[114] and represent the main factors controlling coral species [50], the abundance of artificial habitats and the biological characteristics of *O. patagonica* have played a significant role in the ongoing expansion of this species. In short, the rapid spread process appears to be a response to global change mainly mediated by the interplay of (i) the availability of open space provided by artificial habitats, (ii) seawater temperature increase (mainly by extending the growth period), and (iii) the particular biological features of *O. patagonica* (high current growth rates, early reproduction, and survival to low temperature and in polluted areas).

Despite the evidence that some coral species appear to be responding to climatic warming by expanding their distributions toward the poles ([70], see above), it has been argued that latitudinal migration is unlikely to occur rapidly enough to respond to the current projected temperature change (3–6°C over the next 100 years [124]) due to the significant distance involved (i.e., the latitudinal temperature gradient is ∼1,5°C/1000 kilometers), the effects of temperature on reproduction and the decrease in carbonate ion concentrations at high latitudes [15], [125]–[126]. The present study has characterized the rapid northward expansion of a coral species at high latitudes (40–42°N), a process that has been enhanced by artificial reef structures ahead of the migrating coral. Furthermore, *O. patagonica* is able to reproduce under the environmental conditions at these high latitudes (authors' unpublished data) and even adapt to the effects of repeated bleaching events [34]. Thus, a coral species with particular biological characteristics that allow it to withstand the temperature challenge that accompanies northward migration as well as the natural and anthropogenic side effects that this type of migration involves (i.e., competition with macroalgae, high sediment loads, turbidity, water chemistry) has accomplished a successful northward expansion and may be able to keep pace with the global warming prediction of ∼3°C over the next 100 years.

It is crucial to develop an understanding of the characteristics and the spread rate of a coral that is able to profoundly alter the habitat within which it thrives. In the southeast region of the Iberian littoral zone in the Mediterranean Sea, *O. patagonica* has exhibited a large increase in cover over the last decade (from 3–5% to 10–15%, [24]). Furhermore, this species has been able to induce a persistent phase shift from macroalgal to coral dominance at a particular location [25], which challenges the current conceptual framework [96]. In this evolving context, the rapid northward spread of the species over the last two decades is indicative of an ongoing fundamental modification of temperate shallow water assemblages, which is consistent with predictions [18], [127] that highlight the Mediterranean Sea as one of the ecosystems most sensitive to global change.

## Supporting Information

Text S1 **Sea surface circulation in the Catalan Sea, implications for** ***Oculina patagonica*** **northward dispersal (PDF).**
(PDF)

Figure S1 ***Oculina patagonica*** **. Minimal sampling area for colony density and cover estimates (PDF).**
(PDF)

Figure S2 **Trends on coastal development in the Catalan coast (1970s-2010) (PDF).**
(PDF)

Table S1 Studied locations for sea surface temperature in the Catalan coast (PDF).
(PDF)
