# Supplementary material for: Rapid Northward Spread of a Zooxanthellate Coral Enhanced by Artificial Structures and Sea Warming in the Western Mediterranean
Source: PLoS One. 2013 Jan 14;8(1):e52739. doi: 10.1371/journal.pone.0052739 (PMC3544859; doi:10.1371/journal.pone.0052739)
Supplement: Text S1 — Sea surface circulation in the Catalan Sea, implications for Oculina patagonica northward dispersal (PDF). (PDF) [file pone.0052739.s001.pdf]

Supporting Information

Text S1. Sea surface circulation in the Catalan Sea, implications for *Oculina patagonica* northward dispersal.

The sea surface circulation in the Catalan coast is dominated by a southwestward current (Northern Current, NC) flowing throughout the year along the continental shelf of the French and Catalan coasts (Font *et al.* 1988; Millot 1999). Although the direction of the NC flow is steady southward, its flux is maximum from December to May and its structure markedly changes seasonally. In summer, the NC is relatively wide and tends to flow closer to the coast, whereas in winter it becomes narrower and tends to flow closer to the continental slope (Albérola *et al.* 1995; Sammari *et al.* 1995; André *et al.* 2005). However, the NC displays significant mesoscale structures, mostly eddies and meanders, that affect the circulation at a local scale together with intermittent strong winds from the south (Alvarez *et al.* 1996; Salat *et al.* 2002; André *et al.* 2005). These mesoscale structures undergoes a strong increase in autumn (Font *et al.* 1995; Sammari *et al.* 1995) which may have contributed to the northward dispersion of the species in the opposite direction to the main current. Nevertheless, the permanent southward sea surface circulation of the NC and its flow shoreward shift during the *O. patagonica* spawning and dispersion season in early autumn (Fine *et al.* 2001) is a hydrological factor that may have been diminishing the species rate of northward expansion documented along the Catalan coast.

References

Albérola C, Millot C, Font J (1995) On the seasonal and mesoscale variabilities of the Northern current during the PRIMO 0 experiment in the western Mediterranean Sea. *Oceanologica Acta*, **18**, 163-192.

Alvarez A, Tintoré J, Sabatés A (1996) Flow modification and shelf/slope exchange induced by a submarine canyon off the northeast Spanish coast. *Journal of Geophysical Research*, **101**, 12043-12055.

André G, Garreau P, Garnier V, Fraunié P (2005) Modelled variability of the sea surface circulation in the North-western Mediterranean Sea and in the Gulf of Lions. *Ocean Dynamics*, **55**, 294-308.

Fine M, Zibrowius H, Loya Y (2001) *Oculina patagonica*: A non-lessepsian scleractinian coral invading the Mediterranean Sea. *Marine Biology*, **138**, 1195-1203.

Font J, Salat J, Tintoré J (1988) Permanent features in the circulation of the Catalan Sea. In: *Océanographie Pélagique Méditerranéenne, Oceanologica Acta 9* (eds Minas HJ, Nival P) pp. 51-57. Gauthier-Villars, Paris.

Font J, Garcia-Ladona E, Garcia-Gorriz E (1995) The seasonality of mesoscale motion in the Northern Current of the western Mediterranean: several years of evidence. *Oceanologica Acta*, **18**, 207-219.

Millot C (1999) Circulation in the Western Mediterranean Sea. *Journal of Marine Systems*, **20**, 423-442.

Salat J, Garcia MA, Cruzado A *et al.* (2002) Seasonal changes of water mass structure and shelf-slope exchanges at the Ebro Shelf (NW Mediterranean). *Continental Shelf Research*, **22**, 327-348.

Sammari C, Millot C, Prieur L (1995) Some aspects of the seasonal and mesoscale variabilities of the Northern current inferred from the PROLIG-2 and PROS-6 experiments. *Deep-Sea Research*, **42**, 893-917.
