# Supplementary material for: Rapid Northward Spread of a Zooxanthellate Coral Enhanced by Artificial Structures and Sea Warming in the Western Mediterranean
Source: PLoS One. 2013 Jan 14;8(1):e52739. doi: 10.1371/journal.pone.0052739 (PMC3544859; doi:10.1371/journal.pone.0052739)

Supporting Information

Fig. S1. ***Oculina patagonica.*** **Minimal sampling area for colony density and cover estimates.** Variation of the standard error (SE) as a proportion of the mean with sample size (area in square meters) to determine the minimum sample size for colony density and cover estimates.

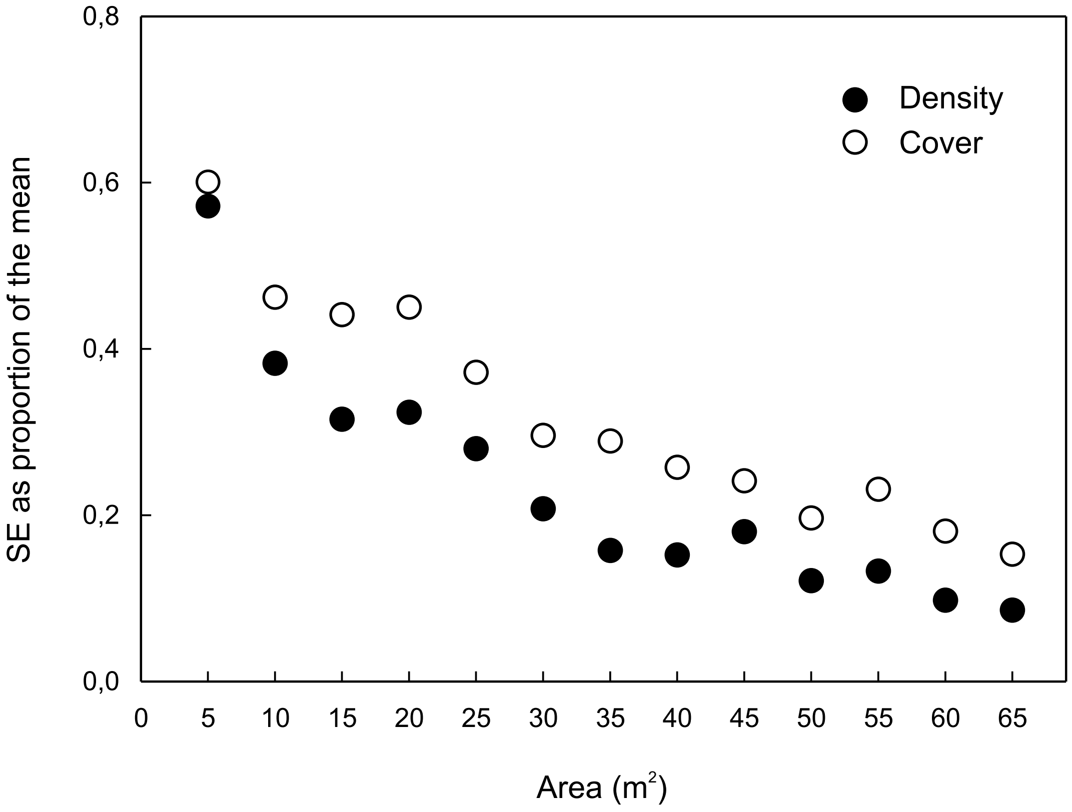

Supplement: Figure S1 — Oculina patagonica . Minimal sampling area for colony density and cover estimates (PDF). (PDF) [file pone.0052739.s002.pdf]
