# Supplementary material for: Rapid Northward Spread of a Zooxanthellate Coral Enhanced by Artificial Structures and Sea Warming in the Western Mediterranean
Source: PLoS One. 2013 Jan 14;8(1):e52739. doi: 10.1371/journal.pone.0052739 (PMC3544859; doi:10.1371/journal.pone.0052739)

Supporting Information

Fig. S2. Trends on coastal development in the Catalan coast (1970s-2010). Modified from Pla de Ports Catalunya 2007-2015 (2007). Generalitat de Catalunya. Departament de Política Territorial i Obres Públiques. ISBN 978-84-393-8417-5 (www.gencat.cat).

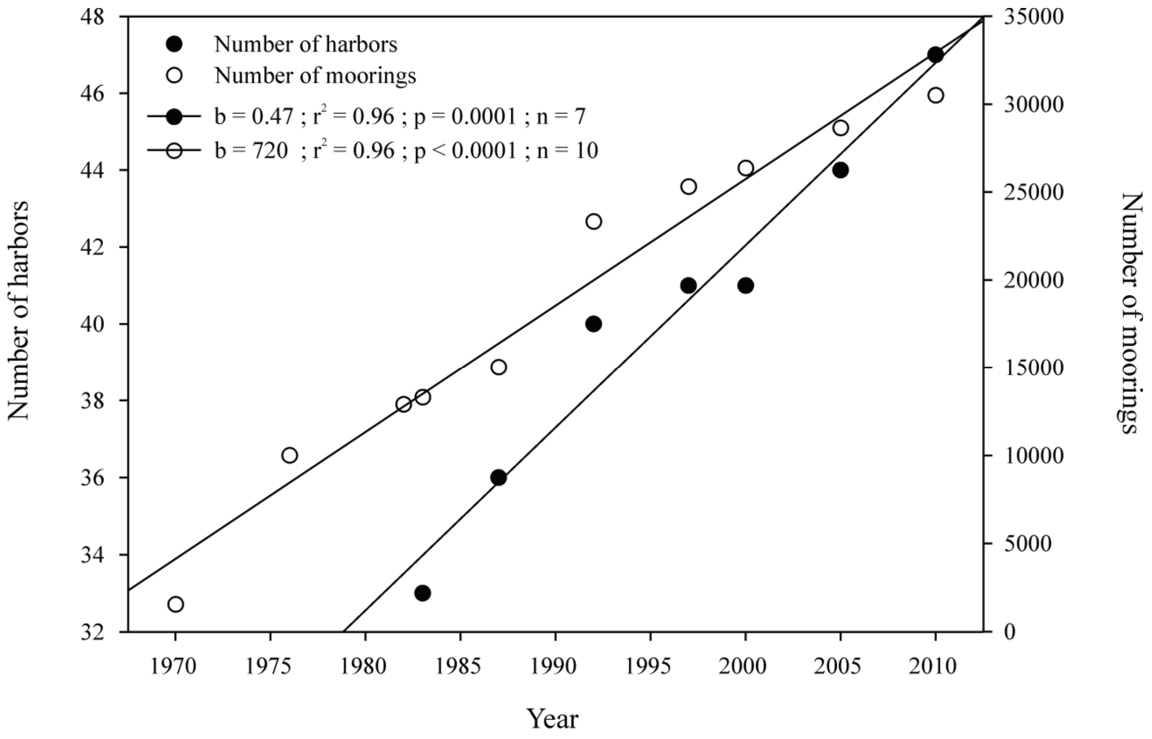

Supplement: Figure S2 — Trends on coastal development in the Catalan coast (1970s-2010) (PDF). (PDF) [file pone.0052739.s003.pdf]
