# Supplementary material for: Rapid Northward Spread of a Zooxanthellate Coral Enhanced by Artificial Structures and Sea Warming in the Western Mediterranean
Source: PLoS One. 2013 Jan 14;8(1):e52739. doi: 10.1371/journal.pone.0052739 (PMC3544859; doi:10.1371/journal.pone.0052739)
Supplement: Table S1 — Studied locations for sea surface temperature in the Catalan coast (PDF). (PDF) [file pone.0052739.s004.pdf]

Supporting Information

Table S1. Studied locations for sea surface temperature in the Catalan coast. The cumulative distances between the locations for which temperature was examined, ordered from south to north, are indicated. N: number of valid daily sea surface temperature (SST) measurements obtained from NASA satellite measurements over the last 8 years (2003–2010).

Zone	Locality	Code	Distance (km) south to north	Geographic coordinates Latitude, N	Longitude, E	SST readings N
1	Les Cases d'Alcanar	CA	0	40°31'05"	0°32'25"	1089
	L'Ampolla	LA	40	40°50'00"	0°46'51"	1202
2	Vilanova i la Geltrú	VG	130	41°11'02"	1°42'05"	1454
	Premià de Mar	PM	200	41°29'28"	2°25'28"	1400
3	Platja d'Aro	PA	280	41°46'47"	3°04'52"	1385
	Medes Islands	MI	325	42°01'14"	3°13'47"	1397
4	Cadaqués	CD	370	42°15'00"	3°18'16"	1410
	Port de la Selva	PS	404	42°21'52"	3°12'19"	1237
